# Effects of a Resistance Exercise Program in Patients with Colorectal Cancer Undergoing Chemotherapy Treatment: A Randomized Controlled Trial Study Protocol

**DOI:** 10.3390/jcm13154478

**Published:** 2024-07-31

**Authors:** Carlos Martin-Sanchez, Eduardo José Fernández-Rodríguez, Yolanda Lopez-Mateos, Alberto Garcia-Martin, Emilio Fonseca-Sanchez, Juan Luis Sánchez-González

**Affiliations:** 1 Department of Nursing and Physiotherapy, Universidad de Salamanca, 37008 Salamanca, Spain; carlos_ms@usal.es (C.M.-S.); juanluissanchez@usal.es (J.L.S.-G.); 2 Institute of Biomedical Research of Salamanca (IBSAL), 37007 Salamanca, Spain; ylopezm@saludcastillayleon.es (Y.L.-M.); efonseca@usal.es (E.F.-S.); 3 Department of Medical Oncology, University Hospital of Salamanca, 37007 Salamanca, Spain; 4 Department of Labour Law and Social Work, University of Salamanca, 37007 Salamanca, Spain; albergm@usal.es

**Keywords:** cancer, protocol, exercise, colorectal cancer

## Abstract

**Simple Summary:**

Colorectal cancer, a major cause of morbidity and mortality, often requires chemotherapy, which can lead to debilitating side effects such as chemotherapy-induced peripheral neuropathy (CIPN). This study aims to evaluate the benefits of a strength exercise program in reducing CIPN in colorectal cancer patients undergoing chemotherapy. Conducted as a double-blind randomized controlled trial with 44 participants, the study divides them into an intervention group (supervised resistance training and home exercises) and a control group (home exercises only). Primary outcomes will be assessed using the EORTC QLQ-CIPN20 questionnaire, while secondary outcomes include quality of life, body mass index, oxygen consumption, anxiety and depression, fatigue, sleep quality, and various blood parameters. The findings will offer valuable insights into how tailored exercise protocols can improve the quality of life, physical capacity, and treatment outcomes for colorectal cancer patients, advocating for the integration of physical exercise into standard cancer care.

**Abstract:**

**Background:** Colorectal cancer is a leading cause of morbidity and mortality worldwide, with chemotherapy being a crucial treatment despite its significant side effects, such as chemotherapy-induced peripheral neuropathy (CIPN). Physical exercise has shown potential benefits in mitigating these side effects and improving patients’ overall well-being. **Objective:** This study aims to evaluate the efficacy of a strength exercise program in reducing CIPN in patients with colorectal cancer undergoing chemotherapy, along with secondary objectives including impacts on quality of life, body mass index, oxygen consumption, anxiety and depression, fatigue, sleep quality, and various analytical parameters. **Methods:** A double-blind randomized controlled trial was conducted with 44 participants, divided into an intervention group (supervised resistance training twice a week and home exercises) and a control group (home exercises only). The primary outcome measure was CIPN, assessed using the EORTC QLQ-CIPN20 questionnaire. Secondary outcomes included assessments using the EORTC QLQ-C30, the 6-minute walk test, HADS, FACT-F, and MISS, along with various blood parameters. **Results and Conclusions:** The study will provide insights into the effectiveness of physical exercise in managing CIPN and improving various health parameters in colorectal cancer patients. By developing tailored exercise protocols, this research aims to enhance patient quality of life, optimize treatment outcomes, and reduce the incidence of debilitating side effects, thereby supporting the integration of physical exercise into standard oncological care.

## 1. Introduction

Colon cancer is one of the leading causes of morbidity and mortality worldwide, with an increasing incidence in many developed countries. In Spain, it is one of the most common types of cancer, accounting for approximately 15% of all cancer cases diagnosed, with an incidence of around 41,000 new cases annually [1]. The mortality associated with colon cancer is significant, although there have been notable improvements in five-year survival rates, which currently stand at around 65%, thanks to advances in treatments and early diagnosis [2]. Early diagnosis is crucial and has been facilitated by screening programs such as colonoscopy and fecal occult blood tests, which allow the detection of precancerous lesions and early-stage cancer. Treatments for colon cancer include surgery, radiotherapy, immunotherapy, and notably, chemotherapy [3]. Chemotherapy is especially relevant in adjuvant treatment to reduce the risk of postoperative recurrence and in metastatic disease to control tumor growth and prolong survival. Modern chemotherapeutic agents, combined with targeted and supportive therapies, have significantly improved treatment outcomes, providing patients with better recovery prospects and an enhanced quality of life during and after treatment.

Chemotherapy is a crucial treatment in the fight against colon cancer, but unfortunately, its impact is not limited solely to cancer cells. Chemotherapeutic agents, designed to target rapidly growing cells, also affect healthy tissues, leading to various side effects. One of the most common adverse effects is peripheral neuropathy, a condition that affects the peripheral nerves and can manifest as sensations of tingling, numbness, pain, or weakness in the limbs [4].

Chemotherapy-induced peripheral neuropathy can be especially challenging for patients with colon cancer, as it further worsens the quality of life during an already difficult period. The nerves responsible for motor and sensory function are compromised, affecting the patient’s ability to perform daily activities and weakening their physical endurance. This phenomenon adds to the emotional and physical burden of battling cancer [5].

However, there are tools that can positively influence these adverse effects, such as physical exercise. Although it may seem contradictory, regular physical exercise has been shown to have beneficial effects on peripheral neuropathy. Physical exercise can improve blood circulation and promote the regeneration of damaged peripheral nerves. Additionally, exercise helps alleviate the pain and discomfort associated with neuropathy, thereby strengthening patients’ functional capacity [6].

Scientific research has demonstrated that physical exercise has multiple benefits for cancer patients, beyond alleviating peripheral neuropathy. Various studies have indicated that regular physical activity can significantly improve immune function, which is crucial for patients undergoing treatments that weaken the immune system. Exercise helps increase the production and efficiency of immune cells, enhancing the body’s ability to fight infections and potentially inhibiting the growth of cancer cells [4].

Furthermore, physical exercise has been associated with a reduction in cancer-related fatigue, one of the most debilitating symptoms experienced by patients during and after treatment. Fatigue can severely limit patients’ ability to engage in daily activities and reduce their quality of life. Research has shown that structured exercise programs, including aerobic and resistance activities, can diminish the sensation of fatigue and improve energy levels and mood, allowing patients to lead more active and fulfilling lives [7].

Physical activity also plays a vital role in weight management and body composition, important factors for cancer survival and recovery. Obesity and overweight are associated with poorer prognosis in many types of cancer, including colon cancer [8]. Regular exercise helps maintain a healthy weight, reduce body fat, and improve muscle mass, which not only contributes to a better quality of life but can also reduce the risk of cancer recurrence and improve overall treatment outcomes. Scientific evidence strongly supports the inclusion of physical exercise as an integral part of the treatment plan for cancer patients [9].

It is essential to highlight that any exercise plan should be tailored to the individual capabilities of each patient, and medical supervision is crucial. Therefore, the combination of chemotherapy and carefully planned physical exercise offers a comprehensive strategy to address both the disease and its side effects, providing colon cancer patients with a better quality of life during their recovery journey.

It is of vital importance to conduct research on the implementation of physical exercise programs in patients with colon cancer to control all the previously mentioned symptoms, such as peripheral neuropathy, fatigue, decreased immune function, and weight management. These investigations should focus on developing personalized and safe exercise protocols that can be effectively integrated into standard oncological treatment [10]. By generating robust evidence on the benefits and best practices for including physical exercise in the management of colon cancer, patient quality of life can be significantly improved, treatment outcomes optimized, and the incidence of debilitating side effects reduced [11]. Moreover, these studies can provide crucial data to guide healthcare professionals in prescribing specific exercise programs, ensuring that patients receive evidence-based interventions that maximize their physical and emotional well-being throughout the recovery process [12].

Therefore, we set the following study objectives. As the main objective, we aim to evaluate the efficacy of a strength exercise program in chemotherapy-induced neuropathy in patients with colorectal cancer undergoing chemotherapy treatment. And as secondary objectives, the following:

1. To assess the impact of a strength exercise program on quality of life in patients with colorectal cancer undergoing chemotherapy treatment.
2. To evaluate the impact of a strength exercise program on body mass index in patients with colorectal cancer undergoing chemotherapy treatment.
3. To evaluate the impact of a strength exercise program on maximum oxygen consumption in patients with colorectal cancer undergoing chemotherapy treatment.
4. To evaluate the impact of a strength exercise program on anxiety and depression in patients with colorectal cancer undergoing chemotherapy treatment.
5. To evaluate the impact of a strength exercise program on fatigue in patients with colorectal cancer undergoing chemotherapy treatment.
6. To evaluate the impact of a strength exercise program on sleep quality in patients with colorectal cancer undergoing chemotherapy treatment.
7. To evaluate the impact of a strength exercise program on analytical parameters (Hb, inflammatory markers, renal function, etc.) in patients with colorectal cancer undergoing chemotherapy treatment.

## 2. Materials and Methods

### 2.1. Study Design and Setting

A double-blind randomized controlled trial was conducted at the Faculty of Nursing and Physiotherapy, University of Salamanca (Spain), following the Consolidated Standards of Reporting Trials (CONSORT) Statement [13]. The current treatment protocol is described according to the recommendations of SPIRIT [14]. The protocol for this trial received approval from the Ethics Committee of the University of Salamanca (record number 1209) and was conducted in accordance with the Declaration of Helsinki. The clinical trial was registered in ClinicalTrials.gov (registration number NCT06404359) (Supplementary File S2, Study flow chart).

### 2.2. Participants and Eligibility Criteria

Participants diagnosed with colorectal cancer undergoing adjuvant chemotherapy treatment were recruited from the outpatient clinics of the Day Oncology Hospital at the University Assistance Complex of Salamanca and from the Asociación Española Contra el Cáncer (AECC).

The inclusion criteria were as follows:

- Individuals diagnosed with colorectal cancer undergoing chemotherapy treatment.
- Sedentary individuals who have not engaged in physical activity in the last 8 weeks.
- Ability to understand assessment tests and perform exercises.

The exclusion criteria:

- Listing contraindications for engaging in physical exercise: Severe musculoskeletal diseases, severe cardiovascular disease, bone metastases, and other conditions as determined by a healthcare professional.
- Patients who need to stop treatment early due to intolerance to treatment.

### 2.3. Interventions

The physical exercise intervention program was specifically designed for colorectal patients undergoing chemotherapy treatment. The training program was focused on resistance exercises, although daily aerobic exercises were also included. The intervention phase will last 12 weeks.

The study sample will be divided into two groups, the intervention group and the control group, detailed below.

#### 2.3.1. Intervention Group

The intervention group will perform a supervised resistance training program two days/week, combined with a physical activity promotion program to be carried out at home three days/week. All the supervised training sessions will be conducted by a physiotherapist in a healthcare center (Supplementary File S1, Comprehensive description of the intervention).

− Supervised training sessions: There will be two weekly sessions, each one lasting 50 min. Each session will have three distinct parts:
  − Warm-up: 10 min of global strength and aerobic exercises at 60% of estimated VO_2_max, focusing on the areas to be primarily targeted during that session.
  − Resistance training: 6 resistance exercises targeting major muscle groups, with a duration of 30 min. Initial resistance will be set at 70% of the estimated 1-RM. When the participant is able to complete three sets of 12 repetitions at the set weight in two consecutive sessions, the weight will be increased by 10%. Sequenced resistance exercise followed in circuit training fashion with 30 s rest period between exercises and 90 s rest period between sets.
  − Cooldown/stretching: 10 min of a combination of breathing exercises and stretching.
  The detailed description of each of the sessions is presented in the Supplementary Materials, which will be repeated in order and cyclically until the 12 weeks of training are completed.
− Physical activity promotion: Participants will complete three weekly home training sessions that will not coincide with supervised training days. Each session will last 20 min, and they will complete two sets of five exercises, with one minute of work and one minute of rest.
  − Day 1
    1. Sitting and standing up from a chair.
    2. Lateral lunge.
    3. Supine bicycle.
    4. Elbow flexion with weights.
    5. Lateral shoulder raises.
  − Day 2
    1. Lunge.
    2. Quadriceps wall isometric.
    3. Glute bridge.
    4. Frontal shoulder raises.
    5. Wall push-ups.
  − Day 3
    1. Walking on tiptoes.
    2. Squats.
    3. Knee-to-chest raises.
    4. Abdominal crunch.
    5. Shoulder press with weights.

Additionally, all participants should walk for 60 min each day of the week.

#### 2.3.2. Control Group

The control group will only perform the physical activity promotion program 5 days/week. This will be combined with walking for 60 min every day of the week.

### 2.4. Outcomes

In the initial assessment, all variables, including sociodemographic factors, will be measured. Subsequently, all outcome variables will be assessed at the end of the 8-week intervention.

Personal and sociodemographic variables are as follows:

- Age (years)
- Sex (male or female)
- Weight (kilograms), height (meters) and body mass index (kg/m^2^)
- Time since diagnosis (years and months)
- Stage of the disease.
- Tumor location (colon or rectum).
- Type of chemotherapy and number of cycles.
- Radiotherapy (yes/no).

#### 2.4.1. Primary Outcomes

The primary outcome measures are chemotherapy-induced peripheral neuropathy (CIPN). Self-reported neuropathy was evaluated using the European Organisation for the Research and Treatment of Cancer Quality of Life (EORTC QLQ) CIPN20 questionnaire, comprising 20 items categorized into three subscales that assess sensory, motor, and autonomic symptoms. Each item was rated on a Likert scale from 1 ‘not at all’ to 4 ‘very much’. Scores were then converted to a 0–100 scale, with higher scores indicating greater symptom severity.

#### 2.4.2. Secondary Outcomes

The secondary outcomes are as follows:

- Quality of life: Assessment will be conducted using the European Organisation for Research and Treatment of Cancer Quality of Life Questionnaire (EORTC QLQ). The EORTC QLQ-C30 is a general quality-of-life instrument for cancer patients [15]. This questionnaire consists of 30 items, 24 of which are grouped into five functional scales (physical, role, emotional, cognitive, and social), three symptom scales (fatigue, pain, and nausea/vomiting), and one global health status scale. The remaining six items assess additional symptoms (dyspnea, appetite loss, insomnia, constipation, and diarrhea) and financial impact. The SELECT BC-CONFIRM trial used the Japanese version of the QLQ-C30 questionnaire (version 3) [16], with each scale/item converted into a score ranging from 0 to 100. A high score on the functional scale indicates greater functional ability, whereas a high score on the symptom scale indicates greater distress. Researchers administered the QLQ-C30 before starting the protocol treatment and every two months thereafter until one year after starting treatment.
- Oxygen consumption: The oxygen consumption will be assessed with the 6-minute walk test (6MWT). The 6MWT was conducted with participants walking freely for 6 min, aiming to cover as much distance as possible along a flat 30 m corridor marked out in meters, adhering to the guidelines set forth by the American Thoracic Society [17]. Upon completion of the test, participants rated their perceived exertion level using the Borg scale [18]. Prior to and after the test, the assessor recorded the participant’s blood pressure in mmHg, heart rate in beats per minute, respiratory rate in breaths per minute, and peripheral oxygen saturation (SpO_2_). Additionally, the total distance covered during the walk was documented.
- Anxiety and depression: Anxiety and depression will be assessed with the Anxiety and Depression Scale (HADS). HADS is a reliable and valid questionnaire [19] for populations, consisting of seven questions (rated 0, 1, 2, and 3) related to anxiety (subscale A) and seven others to depression (subscale D), thus providing two scores.
- Fatigue: Fatigue will be measured by the 13-item Functional Assessment of Cancer Therapy-Fatigue (FACT-F) subscale [20]. FACT-F is a self-reported questionnaire specifically crafted to gauge the degree of fatigue experienced by individuals with cancer and stands as the most frequently employed scale for assessing cancer-related fatigue [21,22]. It comprises 27 items in the FACT-general section and an additional 13 items in the fatigue subscale, with scores ranging from 0 to 160. Higher scores on this scale indicate lower levels of fatigue.
- Quality of sleep: The assessment of insomnia will be conducted using the Minimal Insomnia Symptom Scale (MISS), a concise questionnaire designed for insomnia screening [23]. This tool comprises three items, with responses rated on a five-point Likert scale ranging from 0 (none) to 4 (severe problems). The total score ranges from 0 to 12, with higher scores indicating more significant sleeping difficulties. The reliability and validity of the MISS have been confirmed, particularly among the elderly [23]. In this sample, reliability was measured at 0.79.
- Analytics parameters: A lipid profile and a PCR test will be evaluated.

### 2.5. Sample Size

The sample size calculation was performed using statistical software (G*Power 3.1). The calculation was carried out for a two-independent-group t-test. The effect size (d = 1.11) was estimated from the mean difference on the Chemotherapy-Induced Peripheral Neuropathy (CIPN) Trial Outcome Index (TOI) scale, obtained from Zimmer et al., 2017 [24], due to the similarity of interventions, and from the study by Balnd et al., 2019 [24], where the same scale (EORTC QLQ-CIPN 20) was used in the same group of patients but with a different intervention. A significance level of 0.05 and power of 95% were established. The estimated sample size was 38 subjects. Taking into account a 20% dropout rate based on the previous study [24], the required number of subjects was 44, with 22 subjects in each group.

### 2.6. Allocation and Randomization

To conduct the randomization process in a clinical trial, Microsoft Excel 2020 was used to generate a list of random numbers assigned to the participants. Each participant was given a unique number from the list. Those assigned an odd number were placed in the experimental group, while those assigned an even number were placed in the control group. This method ensures a random and equitable distribution of participants across both groups, minimizing bias and ensuring the validity of the clinical trial results.

### 2.7. Blinding

Due to the nature of the study, participants and evaluators could not be blinded to the intervention. Additionally, the statistical analysis was conducted by an independent statistician who was not aware of the intervention group.

### 2.8. Statistical Methods

For the descriptive analysis of the data, normality will be checked with the Kolmogorov–Smirnov and Shapiro–Wilk tests (*n* < 30). Normally distributed variables will be defined by the mean, standard deviation, and value interval, while variables that do not follow a normal distribution will be defined by median and interquartile range. The qualitative variables of the study will be defined by frequencies and percentages.

Regarding quantitative analysis, a correlation analysis (Pearson’s correlation coefficient) will be used to demonstrate the validity of the assessment procedure selected in our study. Cronbach’s alpha coefficient will be used to demonstrate its reliability. For the comparison of two means, Student’s *t*-statistic inferential analysis (parametric test for independent samples), the Mann–Whitney U-statistic (non-parametric tests with two independent samples), and Wilcoxon’s *t*-test (non-parametric tests for repeated means) will be used. The comparison of three or more means has also been used and will be analyzed by means of an ANOVA test in the situation of independent groups (parametric via Snedecor’s F -ANOVA- and non-parametric via Kruskal–Wallis’ H). For repeated measures, Snedecor’s F test will be used in the parametric way and Friedman’s test in the non-parametric way. Correlations will be carried out by two methods: Pearson’s correlation (normal distribution) or Spearman’s correlation (non-normal distribution). Multivariate logistic regression analysis will be performed to determine the variables associated with events of interest. In the logistic regression model, variables that have been significant in the bivariate analysis or that are relevant to the study will be introduced into the logistic regression model. For qualitative or categorical variables, contingency tables and the Chi-Square test of significance will be used when there are two independent samples.

*p*-values of less than 0.05 will be considered significant, i.e., with a confidence interval of 95%. The statistical software IBM SPSS Statistics version 28.0.1 will be used.

## 3. Discussion

### 3.1. Potential Impact and Significance of the Study

Colorectal cancer is one of the leading causes of morbidity and mortality worldwide. Adjuvant treatments, such as chemotherapy and radiotherapy, are essential for reducing the risk of recurrence, but they are often associated with significant side effects that affect patients’ quality of life. Physical exercise has emerged as a promising complementary intervention that can mitigate these adverse effects, improve physical capacity, and enhance the overall well-being of patients. This physical exercise program is specifically designed to address the unique needs of individuals with colorectal cancer undergoing adjuvant therapy, offering an opportunity to improve both clinical outcomes and patients’ quality of life.

Implementing a physical exercise program can reduce cancer-related fatigue, improve mood, and increase energy levels, contributing to a better quality of life during and after treatment. Additionally, regular exercise can help alleviate some of the most common side effects of adjuvant therapy, such as peripheral neuropathy, muscle loss, and general weakness. Previous studies have indicated that exercise can significantly reduce cancer-related fatigue, as evidenced by research from Mustian et al. (2017) [12], where considerable improvement in the quality of life of cancer patients participating in exercise programs was observed.

A structured exercise program can improve patients’ strength, flexibility, and cardiovascular endurance, allowing them to maintain greater independence and functionality in their daily activities. While research such as that by Courneya et al. (2014) [25] has already shown the benefits of exercise in breast cancer patients, this study will focus specifically on colorectal cancer patients undergoing adjuvant therapy, an area that still requires more targeted research. Although more research is needed, preliminary evidence suggests that exercise may positively influence oncological outcomes, potentially enhancing treatment effectiveness and reducing the risk of cancer recurrence. Studies like those by Meyerhardt et al. (2006) [26] have shown that exercise can be associated with lower recurrence rates and higher survival in colorectal cancer patients, reinforcing the need for further exploration in this area.

Participation in an exercise program can also provide patients with a sense of control and empowerment, improving their psychological and emotional state during treatment. This sense of control can be crucial for the emotional well-being of patients, as highlighted in the study by Pinto and Trunzo (2005) [27], where patients engaged in physical activities reported improved self-esteem and psychological well-being. Furthermore, incorporating physical exercise as part of the treatment regimen can promote long-term healthy lifestyle habits, which can have sustained benefits for patients’ overall health. Previous research, such as that by Schmitz et al. (2010) [28], has demonstrated that patients who adopt an active lifestyle during and after treatment have better quality of life and fewer long-term health complications.

In summary, this physical exercise program has the potential to offer significant benefits to individuals with colorectal cancer undergoing adjuvant therapy, improving their quality of life, physical capacity, and possibly their clinical outcomes, while promoting a positive attitude and healthy lifestyle habits. By comparing the expected findings of this study with existing literature, it is evident that this research will significantly contribute to the current knowledge base, providing specific data on the effects of exercise in colorectal cancer patients. This will not only help improve current treatment strategies but also offer valuable guidance for future research and clinical practices in this field.

### 3.2. Limitations

For the study, we identified two main limitations. First, the potential comorbidities of the individuals participating in the sample, whether pre-existing before the pathoanatomical diagnosis of cancer or secondary to the tumor process and its treatments, which could influence the standardization of interventions. Second, the progression of the disease itself, as a negative progression or changes in the tumor process could result in the loss of some study subjects. Nonetheless, we consider both limitations to be normal in any complex clinical process.

### 3.3. Ethics and Dissemination

The study is being conducted in accordance with the protocol and principles of the current version of the Declaration of Helsinki [29]. Permission to conduct the study was granted by the Ethics Committee of the University of Salamanca in May 2024 (ref: 1209).

All patients participating in the study will be informed about the study’s objectives and will provide their written informed consent. The patients’ right to privacy will be respected, applicable data protection laws will be followed, and the anonymity of all study participants will be guaranteed when data are published in scientific journals. The medical data of the patients will be considered confidential and will not be disclosed to third parties. To ensure proper confidentiality, identification numbers will be randomly assigned to each participant. Therapeutic conditions will be tailored to each patient, with modifications made as necessary to ensure patient safety and provide the most individualized treatment possible. The risk of therapy-related adverse events is minimal.

However, if serious adverse events occur during or after the study, they will be monitored by the study team and physicians as part of standard therapy. All serious adverse events will be documented and reported immediately (within a maximum of 24 h) to the principal investigator of the study.

The study will respect the rights of participating patients and ensure the confidentiality of their information. Participants will be assigned a specific trial number to identify their data. Personal details of participants will be stored separately at each site under the guidelines of the 1988 Data Protection Act and will not be entered into the trial database. Personal data will not be retained longer than necessary for the purposes for which it was collected.

## Data Availability

The original data presented in the study will be openly available in the GREDOS repository of the University of Salamanca.

## Supplementary Materials

The following supporting information can be downloaded at: https://www.mdpi.com/article/10.3390/jcm13154478/s1, Supplementary File S1, Comprehensive description of the intervention. Supplementary File S2, Study flow chart.
