# Supplementary material for: Effects of a Resistance Exercise Program in Patients with Colorectal Cancer Undergoing Chemotherapy Treatment: A Randomized Controlled Trial Study Protocol
Source: J Clin Med. 2024 Jul 31;13(15):4478. doi: 10.3390/jcm13154478 (PMC11313390; doi:10.3390/jcm13154478)
Supplement: Supplementary file 1 [file jcm-13-04478-s001.zip › jcm-3109596-supplementary-S1.pdf]

Suplementary Materal 1. Comprehensive description of the intervention.

SESSION 1

1.- Warm-up (10 minutes)

- 2 minutes: Walk in circles taking advantage of the width of the room incorporating mobility exercises.
- 20 seconds: Just walk.
- 20 seconds: Shoulder circles forward.
- 20 seconds: Shoulder circles backward.
- 20 seconds: Alternating knee lifts to chest.
- 20 seconds: Walk on tiptoes.
- 20 seconds: Hip separation walking.
- 2 minutes: Single-leg balance with slight knee and hip flexion, 1 minute on each leg.
- 1 minute: Wall/floor push-ups.
- 1 minute: Wall sit.
- 2 minutes: Skipping.
- 2 minutes: Ball toss in pairs or against a wall.

2.- Resistance Training

- Normal squat.
- Romanian deadlift.
- Abdominal plank (30 seconds).
- Bench press with barbell.
- Bicep curls with dumbbells.
- Dumbbell rows.

**There will be 6 stations, and participants will rotate from one to the next until completing all 6 exercises. They will do 3 sets of 12 repetitions at 70% of 1RM. Rest 30 seconds between stations and at the end of each complete set, rest for 90 seconds.*

3.- Cool-down

- 3 minutes: Slow-paced walking with diaphragmatic breathing.
- 1 minute: Quadriceps stretch.
- 1 minute: Hamstring stretch.
- 1 minute: Calf stretch.
- 1 minute: Bicep stretch.
- 1 minute: Shoulder stretch.
- 2 minutes: Seated cervical mobility exercises.

SESSION 2

1.- Warm-up (10 minutes)

- 2 minutes: Walk in circles taking advantage of the room's width incorporating mobility exercises.
- 20 seconds: Just walk.
- 20 seconds: Shoulder abduction/adduction in transverse plane.
- 20 seconds: Alternating shoulder flexion/extension.
- 20 seconds: Leg lifts with straight knees alternately.
- 20 seconds: Heel walks.
- 20 seconds: Jump in place.
- 2 minutes: Ankle proprioception on single-leg support, 1 minute on each leg.
- 1 minute: Knee lift with contralateral arm raise.
- 1 minute: Bodyweight squats/sit and stand from a chair.
- 2 minutes: Step-up with contralateral knee raise.
- 2 minutes: Boxing (punching forward with alternate arms at a slow pace).

2.- Resistance Training

- Lunge.
- Glute bridge with barbell/kettlebell.
- Abdominal crunches.
- Dumbbell bench press.
- Shoulder press.
- Sit-to-stand jumps.

**There will be 6 stations, and participants will rotate from one to the next until completing all 6 exercises. They will do 3 sets of 12 repetitions at 70% of 1RM. Rest 30 seconds between stations and at the end of each complete set, rest for 90 seconds.*

3.- Cool-down

- 3 minutes: Slow-paced walking with diaphragmatic breathing.
- 1 minute: Quadriceps stretch.
- 1 minute: Glute stretch.
- 1 minute: Adductor stretch.
- 1 minute: Hamstring stretch.
- 1 minute: Shoulder stretch.
- 2 minutes: Seated cervical mobility exercises.

SESSION 3

1.- Warm-up (10 minutes)

- 2 minutes: Walk in circles taking advantage of the room's width incorporating mobility exercises.
- 20 seconds: Just walk.
- 20 seconds: Elbow flexion/extension.
- 20 seconds: Shoulder abduction/adduction.
- 20 seconds: Hip flexion + abduction.
- 20 seconds: Walking lunges.
- 20 seconds: Side steps.
- 2 minutes: Lateral step-ups, 1 minute on each leg.
- 1 minute: Close-grip push-ups on wall/floor.
- 1 minute: Bodyweight alternating lunges in place.
- 2 minutes: Jumping jacks.
- 2 minutes: Bodyweight glute bridges.

2.- Resistance Training

- Wide stance squat.
- Deadlift.
- Barbell rows.
- Barbell bicep curls.
- Dumbbell lateral raises.
- Single-leg box step-ups.

**There will be 6 stations, and participants will rotate from one to the next until completing all 6 exercises. They will do 3 sets of 12 repetitions at 70% of 1RM. Rest 30 seconds between stations and at the end of each complete set, rest for 90 seconds.*

3.- Cool-down

- 3 minutes: Slow-paced walking with diaphragmatic breathing.
- 1 minute: Quadriceps stretch.
- 1 minute: Glute stretch.
- 1 minute: Hamstring stretch.
- 1 minute: Bicep stretch.
- 1 minute: Shoulder stretch.
- 2 minutes: Seated cervical mobility exercises.

SESSION 4

1.- Warm-up (10 minutes)

- 2 minutes: Walk in circles taking advantage of the room's width incorporating mobility exercises.
- 20 seconds: Just walk.
- 20 seconds: Squat and stand up as you walk.
- 20 seconds: Trunk rotations with shoulder push.
- 20 seconds: Shoulder raises.
- 20 seconds: Side lunges.
- 20 seconds: Walk backward.
- 2 minutes: Sit and stand from a chair.
- 1 minute: Theraband elbow flexion.
- 1 minute: Calf raises and holds statically.
- 2 minutes: Jumping in place.
- 2 minutes: Shoulder circumduction with light weight.

2.- Resistance Training

- Lateral lunge.
- Deadlift.
- Incline bench chest fly with dumbbells.
- Supine tricep press.
- Front shoulder raises with plate.
- Jump squats.

**There will be 6 stations, and participants will rotate from one to the next until completing all 6 exercises. They will do 3 sets of 12 repetitions at 70% of 1RM. Rest 30 seconds between stations and at the end of each complete set, rest for 90 seconds.*

3.- Cool-down

- 3 minutes: Slow-paced walking with diaphragmatic breathing.
- 1 minute: Quadriceps stretch.
- 1 minute: Glute stretch.
- 1 minute: Tricep stretch.
- 1 minute: Chest stretch.
- 1 minute: Shoulder stretch.
- 2 minutes: Seated cervical mobility exercises.
