# Supplementary figures and images for: Effects of a Resistance Exercise Program in Patients with Colorectal Cancer Undergoing Chemotherapy Treatment: A Randomized Controlled Trial Study Protocol
Source: J Clin Med. 2024 Jul 31;13(15):4478. doi: 10.3390/jcm13154478 (PMC11313390; doi:10.3390/jcm13154478)

## S2. Study flow chart

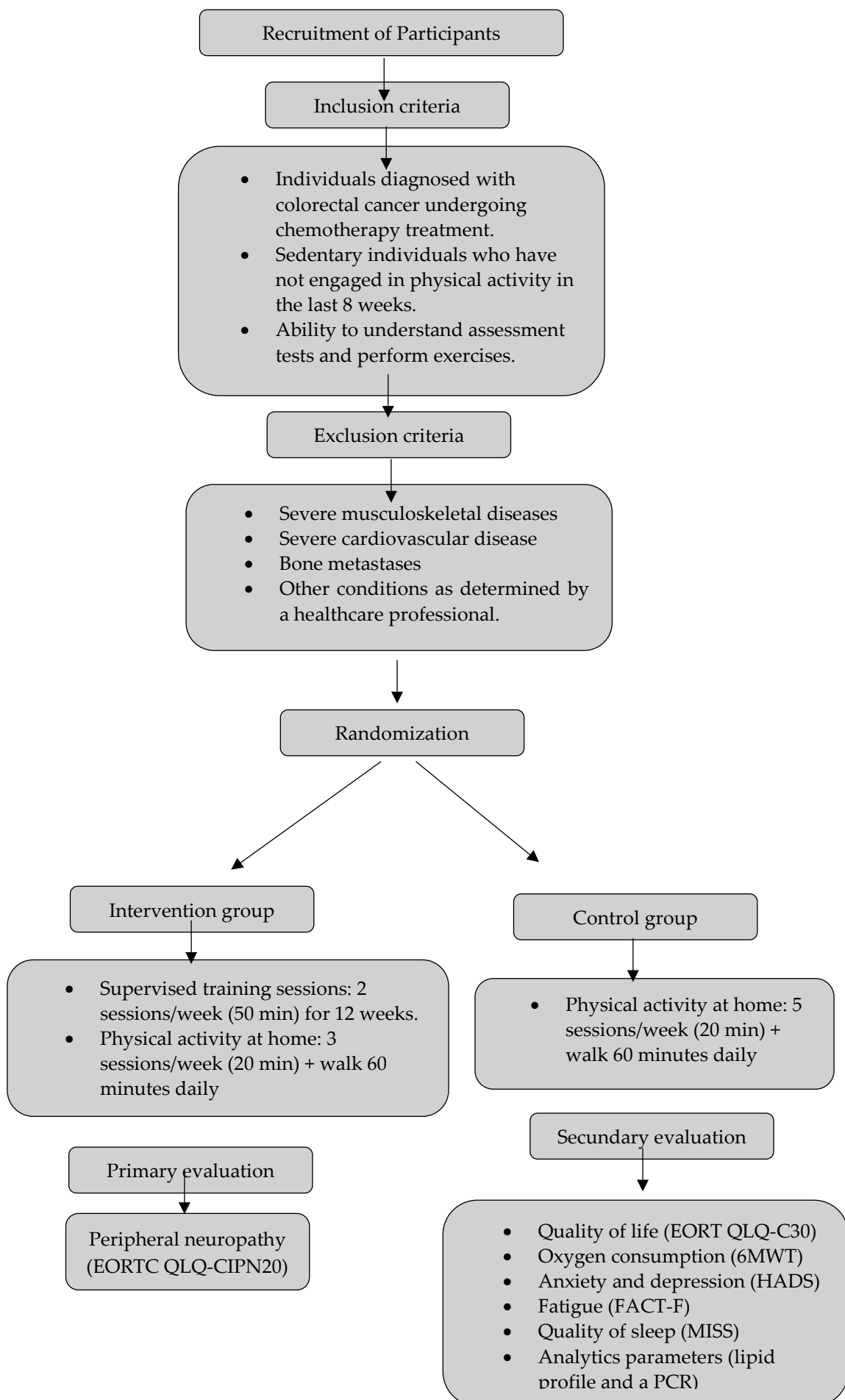

Supplement: Supplementary file 1 [file jcm-13-04478-s001.zip › jcm-3109596-supplementary-S2.pdf]
